# Phaeochromocytomas overexpress insulin transcript and produce insulin

**DOI:** 10.1530/EC-21-0269

**Published:** 2021-06-25

**Authors:** Ivar Følling, Anna B Wennerstrøm, Tor J Eide, Hilde Loge Nilsen

**Affiliations:** 1 Department of Endocrinology, Akershus University Hospital, Lørenskog, Norway; 2 Institute of Clinical Medicine, University of Oslo, Oslo, Norway; 3 Department of Clinical Molecular Biology, University of Oslo and Akershus University Hospital, Lørenskog, Norway; 4 Division of Laboratory Medicine, Department of Pathology, Oslo University Hospital, Oslo, Norway

**Keywords:** phaeochromocytoma, insulin, *INS-IGF2*, ectopic production, ectopic transcription, tumour-induced hypoglycaemia, tumour-induced hyperglycaemia, immunohistochemistry

## Abstract

**Introduction:**

Phaeochromocytomas are tumours originating in the medulla of the adrenal gland. They produce catecholamines, and some tumours also produce ectopic hormones. Two types of glucose imbalances occur in phaeochromocytoma patients, hyperglycaemia and hypoglycaemic attacks. Therefore, we tested whether insulin transcript (*INS*), insulin, and a hybrid read-through transcript between exons from insulin and insulin-like growth factor 2 (*INS-IGF2*) were expressed in phaeochromocytomas.

**Methods:**

We measured the expression of insulin using immunohistochemistry. The expression of *INS-IGF2* was determined by qRT-PCR in formalin-fixed and paraffin-embedded tissue from 20 phaeochromocytomas. The expression of *INS* and *INS-IGF2* transcriptswas also analysed in 182 phaeochromocytomas and paragangliomas using publicly available datasets in The Cancer Genome Atlas (TCGA) Database.

**Results:**

Of 20 phaeochromocytomas, 16 stained positive for insulin. The distribution of positive cells was mostly scattered, with some focal expression indicating clonal expansion. Nineteen tumours expressed high levels of *INS* and *INS-IGF2* transcripts. The expression of the two transcripts corresponded closely. In the TCGA dataset, phaeochromocytoma expresses higher levels of *INS* and *INS-IGF2* transcripts compared to the normal non-tumour adrenal glands. Thus, the expression of *INS* and *INS-IGF2* seems to be a general phenomenon in phaeochromocytoma.

**Conclusion:**

Most phaeochromocytomas contain cells that overexpress *INS* and *INS-IGF2* transcripts. Most tumours also display heterogeneous expression of polypeptides immunoreactive to monoclonal anti-insulin antibodies. Clinically this may relate to both hyperglycaemia and hypoglycaemic attacks seen in patients with phaeochromocytoma as well as autocrine tumour growth.

## Introduction

Phaeochromocytomas are tumours originating in the medulla of the adrenal gland. As the normal adrenal medulla, phaeochromocytomas mainly produce catecholamines but a few also produce ectopic hormones. Clinically, the most important ectopic hormone is adrenocorticotropic hormone causing Cushing syndrome that adds to the typical phaeochromocytoma symptoms (1, 2). Staining with anti-insulin was described in five of seven phaeochromocytomas (3).

Other examples of ectopic insulin production are rare. It is found in a few cases with tumours in the cervix, lung, kidney, paraganglioma, and ovary. The evidence for insulin production in these tumours is, for some of them, limited to staining with anti-insulin antibodies (4, 5, 6, 7). Other studies, in addition, include *in situ* hybridisation of insulin mRNA (8), insulin extracted from tumour (9), cultured cells demonstrating insulin production (10), granules similar to those in beta cells (11, 12), and/or cure of hypoglycaemia after tumour extirpation (10, 12, 13). There are also reports of patients with hyperinsulinemic hypoglycaemia without convincing evidence that their extrapancreatic tumour produced the insulin.

Dysregulated ectopic production of insulin and related molecules could cause or contribute to the two kinds of glucose imbalances seen in patients with phaeochromocytoma. First, hyperglycaemia is common, occurring in 21–37% of the patients and is probably caused by the secreted catecholamines that inhibit insulin secretion and increase insulin resistance (14). In addition, we suggest that insulin-related molecules partly mimicking insulin may block the insulin receptor, and thereby contribute to hyperglycaemia. Secondly, rare cases with hypoglycaemic attacks have been reported in phaeochromocytoma patients (15, 16, 17, 18) but the mechanism is unknown. Hypersecretion of insulin from the tumour may cause hypoglycaemia.

**Figure 1:**
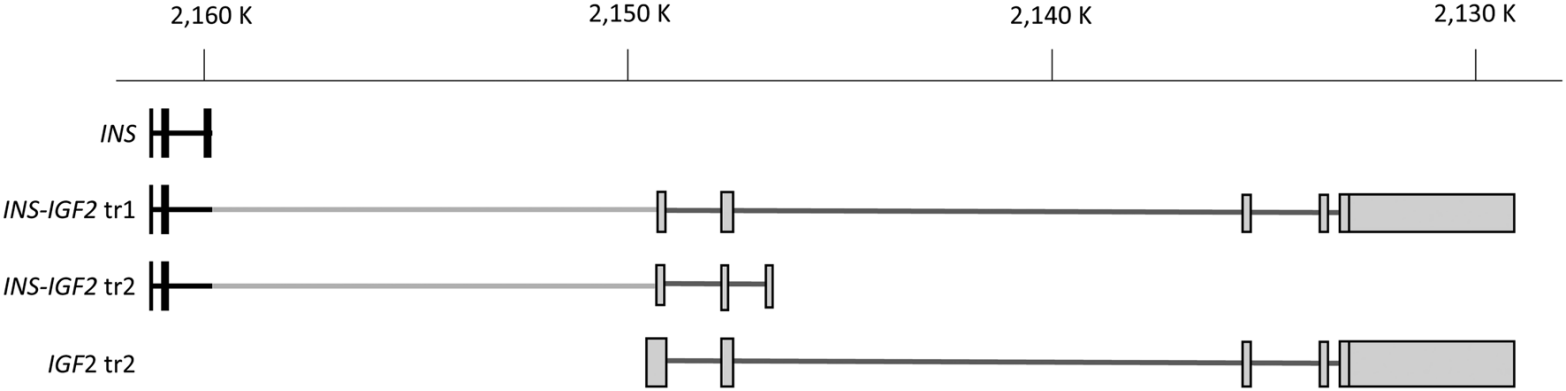
Overview of the transcripts generated from the *INS-IGF2* locus on chromosome 11. Starting from the top, the four transcripts shown are the *INS* transcript, the long *INS-IGF2* transcript 1 (noncoding), the short *INS-IGF2* transcript 2 (coding), and the *IGF2* transcript 2. Exons are depicted as tall boxes and intron as line. Parts annotated as encoded within the insulin gene are shown in black, and the light grey parts designate *IGF2* gene origin. The scale above indicates the genomic position.

Phaeochromocytomas overexpress insulin-like growth factor 2 transcript (*IGF2* transcript) (19) and the translated protein IGF2 (20). The insulin gene and the *IGF2* gene are located after each other on chromosome subband 11p15.5 (Fig. 1). These two genes can give rise to read-through hybrid transcripts composed of exons from both genes: the *INS-IGF2* transcript 1, a long transcript believed never to be translated into protein as an early stop codon targets it to nonsense-mediated decay, and *INS-IGF2* transcript 2, a shorter transcript (Fig. 1) that codes for a 200 amino acid protein.

In this study, we report that most phaeochromocytomas overexpress *INS* and *INS-IGF2* transcripts and insulin.

## Methods

### Patients

We obtained consent from 20 patients with phaeochromocytomas operated consecutively from 2015 to 2017, to retrospectively investigate their tumours. There were an equal number of female and male (10/10) patients with age span 30–70 years (median 57). Two patients had known germline mutation predisposing to phaeochromocytoma; one heterozygote FN1 mutation: c.1527+4_1527+7 del and one heterozygote MAX mutation: exon 3 c.149T>A (Val 50 Asp). Seven patients had hyperglycaemia. Seventeen of the 20 patients were Caucasians. No patients showed evidence of predisposing germline mutations in SDH genes. The hospital recruits unselected phaeochromocytoma patients (Supplementary Table 1, see section on supplementary materials given at the end of this article). All data in the table are as expected in an average cohort of phaeochromocytoma patients. Consent was given by the Regional Ethics committee (2018/196) and The Data Protection Officer (7_2018).

### Tumours

Neighbouring slices of formalin-fixed paraffin-embedded (FFPE) tissue from the 20 tumours were used for RNA studies and immunochemistry. Tumour diameters were 1.9–9.5 cm (median 5 cm).

### Total RNA extraction

Slices of 10 µm were mounted on glass slides and left to dry overnight at room temperature. Macrodissection of tumour tissue was performed to obtain pure tumour tissue cleared of the normal adrenal cortex. Total RNA was extracted using the RNeasy FFPE Kit (Qiagen) as recommended by the manufacturer, and RNA concentration was measured with Qubit RNA BR kit (Invitrogen). RNA quality was measured using Bioanalyzer, and the DV200 values were calculated to be >85% for phaeochromocytoma samples and the negative control and 39% for the positive control. Thus, all samples meet the standard 30% cut-off.

### PCR and quantitative reverse transcriptase PCR (RT-qPCR)

Expression of insulin transcripts was detected with RT-qPCR. For cDNA synthesis, 300 ng of total RNA was reverse transcribed with SuperScript VI, Random Hexamers, dNTP, RNaseOUT RNase Inhibitor (all from Invitrogen), as recommended by the manufacturer. With cDNA input equivalent to 4 ng total RNA, using Power SYBR Green PCR Master Mix (Applied Biosystems) with primers F: 5’-GAAGCGTGGCATTGTGGAA-3’ and R: 5’- GCGTCTAGTTGCAGTAGTTCT-3’. The PCR programme (10 min at 95°C, 30/34× (15s 95°C, 1min 62°C)) was run on Mastercycler ×50 (Eppendorf, Hamburg, Germany). For the *INS-IGF2* transcript 2, we used QuantiTech Primer Assay (Qiagen) using PCR conditions as described for insulin. In all reactions, normal pancreas and normal adrenal gland were used as controls.

PCR products were separated by electrophoresis on 2% TAE/agarose gels using UltraPure Agarose (Invitrogen). Pictures were acquired and quantified by fluorescence using the Gel-doc XR+ system and ImageLab 5.2.1 software (both from Bio-Rad).

Relative quantification of insulin expression was also performed by qPCR, by comparing the Ct value from patient samples with Ct values from a standard curve made from dilutions of the insulin cDNA from the pancreas. The reaction was run in a 384 format on QuantStudio7 (Applied Biosystems), otherwise as described for regular PCR.

### Bioinformatics

Gene expression data from pheochromocytoma and paraganglioma (PCPG) are available through the Cancer Genome Atlas (TCGA) Research Network (https://www.cancer.gov/about-nci/organization/ccg/research/structural-genomics/tcga). The PCPG cohort consists of data from 182 tumours.

The differential gene expression analysis compared expression (cut off value of 1.5) in the PCPG tumours with the normal controls was performed by ANOVA analysis in the online analysis tools Gene Expression Profiling Interactive Analysis (GEPIA) (21).

### Immunohistochemistry

Formalin-fixed and paraffin-embedded slices of 2 µm thick were stained with monoclonal mouse anti-human insulin NCL-INSULIN, clone 2D11-H5, dilution 1:100 (Leica Biosystems) and visualised by chromogen detection using UltraView DAB with amplification (Ventana, Tuscon, USA). Semi-quantification of staining (score 0–4) based on the percentage of stained cells per microscopic visual field with 20× objective: score 0, no positive cells; score 1, 1–25% cells; score 2, 26–50% cells; score 3, 51–75% cells and score 4, >75% cells. The scoring was performed twice, blinded, and with identical results.

Staining with antibodies to succinate dehydrogenase B (SDHB) was performed to assess SDH deficiency (22).

We obtained the RNA results without the knowledge of the immunohistochemistry results and vice versa.

## Results

### Phaeochromocytomas express insulin and hybrid transcripts

**Figure 2:**
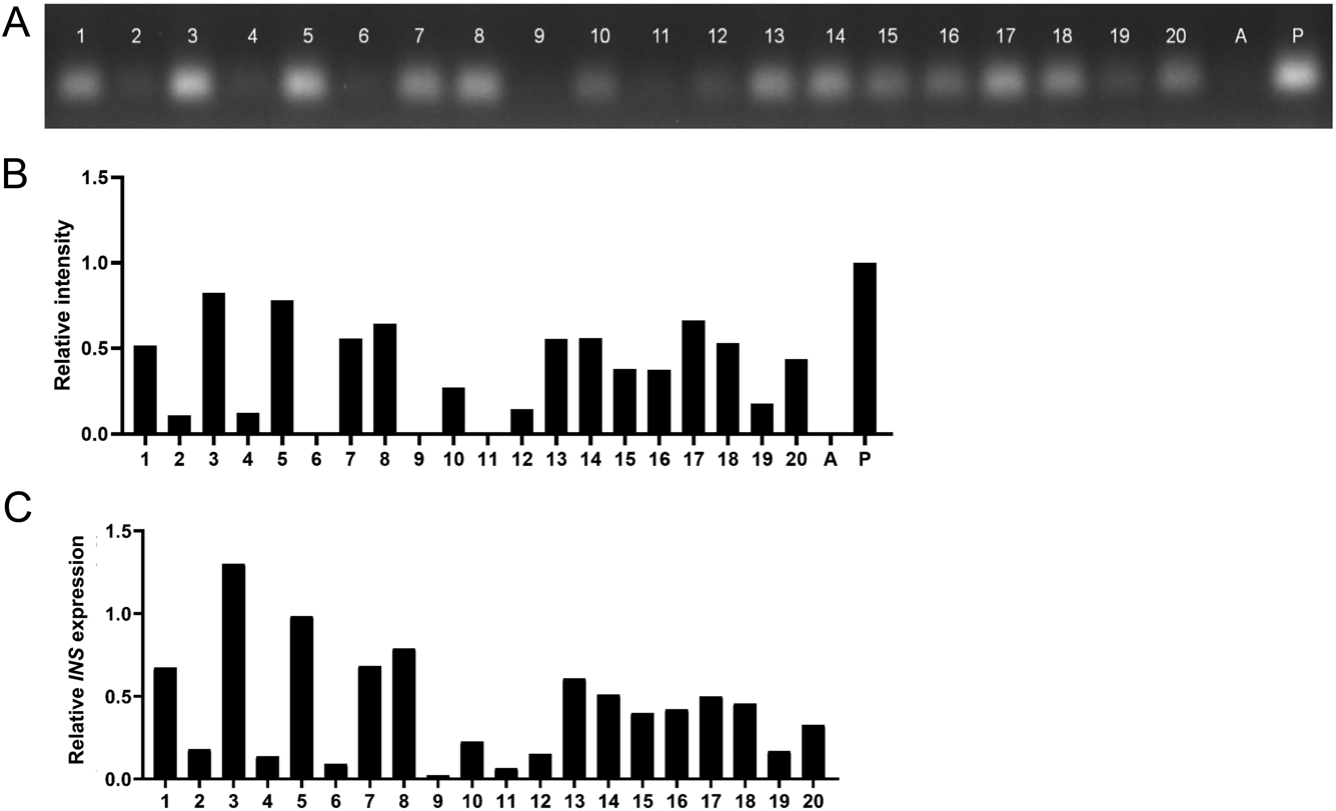
Phaeochromocytomas express insulin transcripts. (A) Agarose gel showing the insulin PCR product after 30 cycles of PCR starting with the equivalent of 4 ng RNA. Of the 20 phaeochromocytoma samples tested (lanes 1–20), 17 show a band indicating amplification of *INS* transcripts. Adrenal gland (lane a) and pancreas (lane p) were used as a negative and positive control, respectively. (B) The relative quantification of signal intensity in the bands in (A) is shown in the bar diagram. The pancreas control was set to 1.0. (C) *INS* expression analysed with qRT-PCR. Total RNA from pancreatic tissue was reverse transcribed to cDNA and serially diluted to form a reference curve. The equivalent of 4 ng total RNA from the phaeochromocytoma sample was used, and the resulting threshold cycle was related to the pancreatic cDNA dilution curve. With this more sensitive method, two more tumours (6 and 11) showed expression of *INS* transcript.

While normal adrenal tissue showed no detectable expression of *INS* transcripts, 17 of the 20 tumours had detectable expression (Fig. 2A). The levels varied from 11 to 83% of normal pancreatic tissue based on quantification of the amplified PCR products separated by agarose gel electrophoresis (Fig. 2A). To get a more sensitive measurement of the expression levels, we performed qRT–PCR analysis where we related the *INS* transcript expression levels to a dilution curve of pancreatic cDNA. The relative order of high to low expression samples did not change substantially, but with the more sensitive method, we detected the expression of insulin in two additional samples, samples 6 and 11 (Fig. 2B), giving detectable expression of *INS* transcript in 19 of 20 tumours.

**Figure 3:**
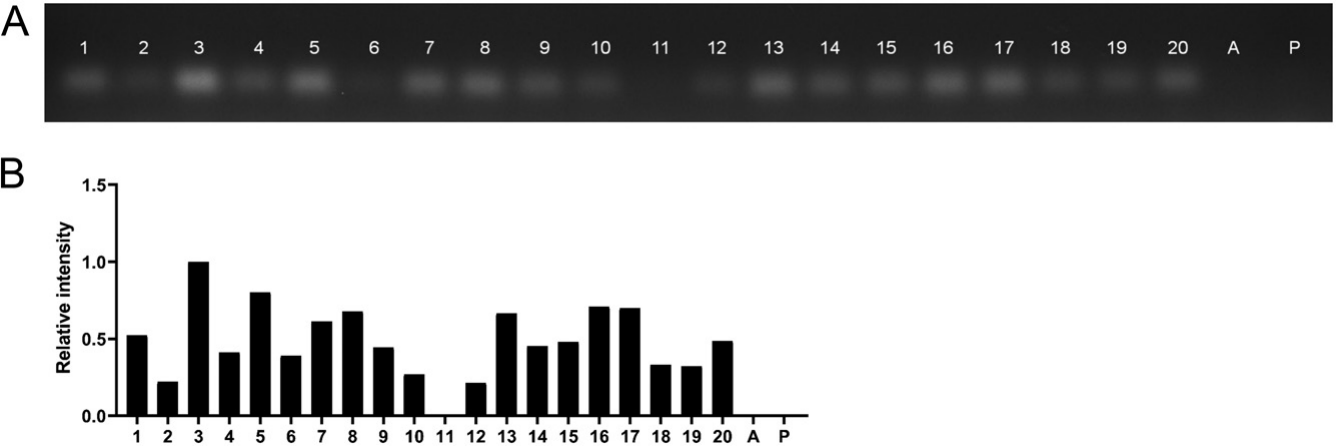
Phaeochromocytoma expresses *INS-IGF2* transcript 2. (A) Agarose gel showing the *INS-IGS2* transcript 2 PCR product of the expected size. Adrenal gland (lane a) and pancreas (lane p) were included as controls. (B) The relative quantification of signal intensity in the bands in (A) is shown in the bar diagram. The highest expressed sample was set as 1.0. The results correlate with the *INS* expression shown in Fig. 2 (Spearman, *r* = 0.80, *P* < 0.002).

As the *INS* gene is located in the upstream of IGF2, we also amplified the hybrid *INS-IGF2* transcript 2 (Fig. 3). An *INS-IGF2* transcript was found in 19 of the 20 tumours. The PCR reaction for *INS-IGF2* transcript needed four more cycles to produce a visible band on the gel, indicating lower levels of *INS-IGF2* than *INS* transcripts. There was no expression of *INS-IGF2* transcript 2 in normal adrenal and pancreatic tissue. Hence, of the 20 phaeochromocytomas, 19 expressed the *INS* transcript and the hybrid *INS-IGF2* transcript 2. Their degree of expression of insulin and hybrid transcripts corresponded closely, indicating transcriptional activation of the whole region around the insulin gene.

### Phaeochromocytomas with different degrees of anti-insulin antibody staining

**Figure 4:**
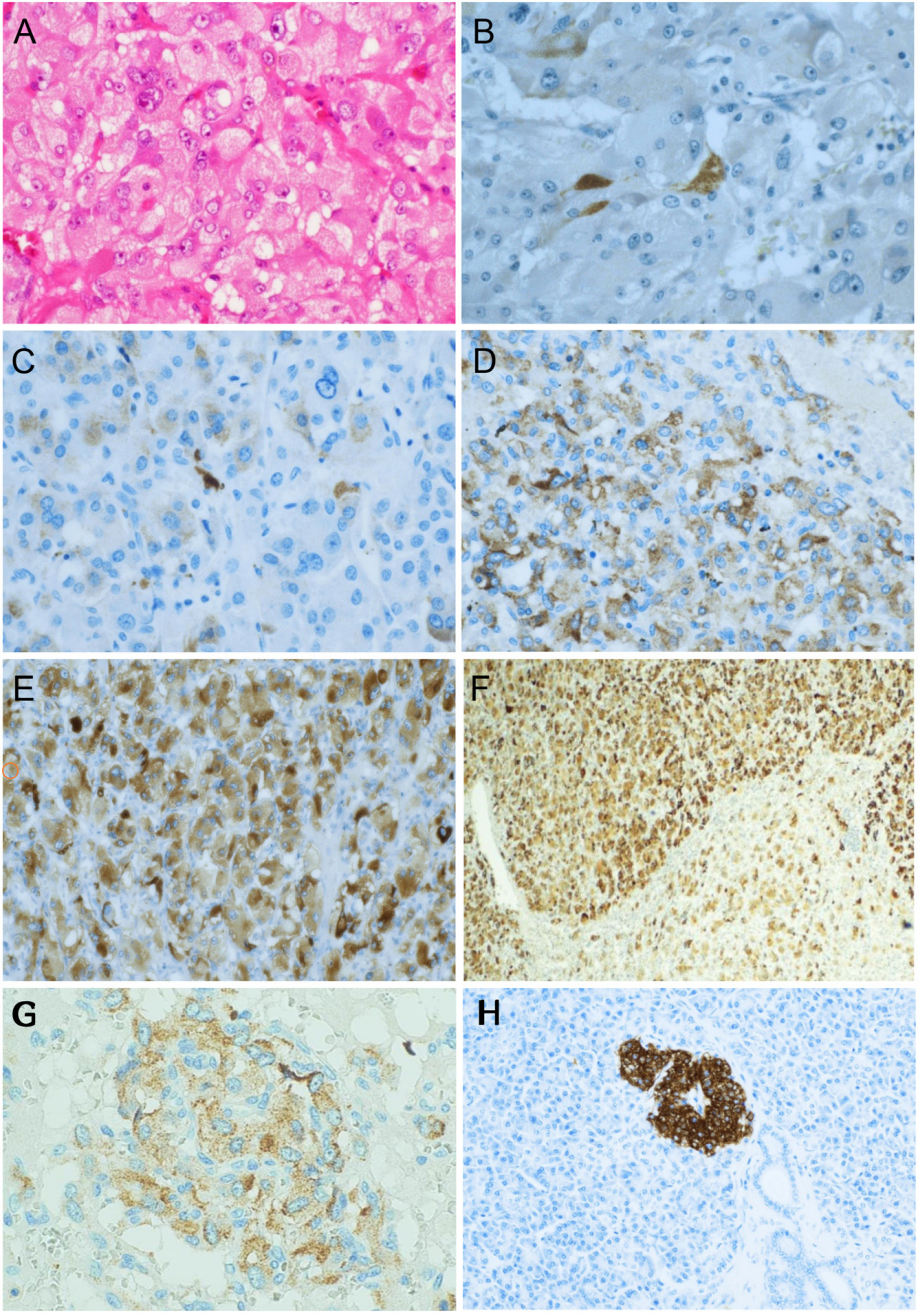
Phaeochromocytomas with different degrees of anti-insulin antibody staining. (A) Standard haematoxylin-eosin staining of phaeochromocytoma. (B, C, D and E) Representative images of degree of positive staining, score 1–4: (B) score 1, 0–25% positive cells, (C) score 2, 26–50% positive cells, (D) score 3, 51–75% positives cells, (E) score 4, >75% positive cells. (F) Large clone (upper part) with heavily stained cells (score 4). (G) Cluster of stained cells, indicating a small clonal distribution of positive cells. (H) Normal pancreas with stained *β*-cells (positive control) surrounded by unstained exocrine cells (negative control).

We used immunohistochemistry to verify whether the transcripts were translated into the corresponding polypeptides. Staining with anti-insulin antibodies was mostly at a low level (score 1, twelve tumours). Four positive tumours showed a higher level of staining (score 2, one tumour; score 3, two tumours; score 4, one tumour); representative examples in Fig. 4B, C, D and E. Only four tumours showed no positive staining.

The distribution of positive cells seems to be mostly scattered (Fig. 4B, C, D and E). However, in one sample, there was an expanding clone of strongly positive cells (Fig. 4F), and in some other samples, we found clusters of positive cells (Fig. 4G). The intracellular-stained granules in positive cells (Fig. 4G) were similar to insulin granules in *β*-cells. We found no staining of control tissues from normal liver, colon, lymph node, adrenal cortex and medulla (not shown), and exocrine pancreas (Fig. 4H).

These results show that of the 20 phaeochromocytomas 16 stained positively with anti-insulin antibodies, indicating translation of the transcripts into polypeptides. The clonal distribution indicates that different parts of a tumour show different transcriptional activation leading to different *INS* and *INS-IGF2* expression and corresponding polypeptide synthesis. This is in line with the established concept of tumour heterogeneity.

### Expression of *INS* and *INS-IGF2* transcripts in phaeochromocytoma tumours validated in TCGA

**Figure 5:**
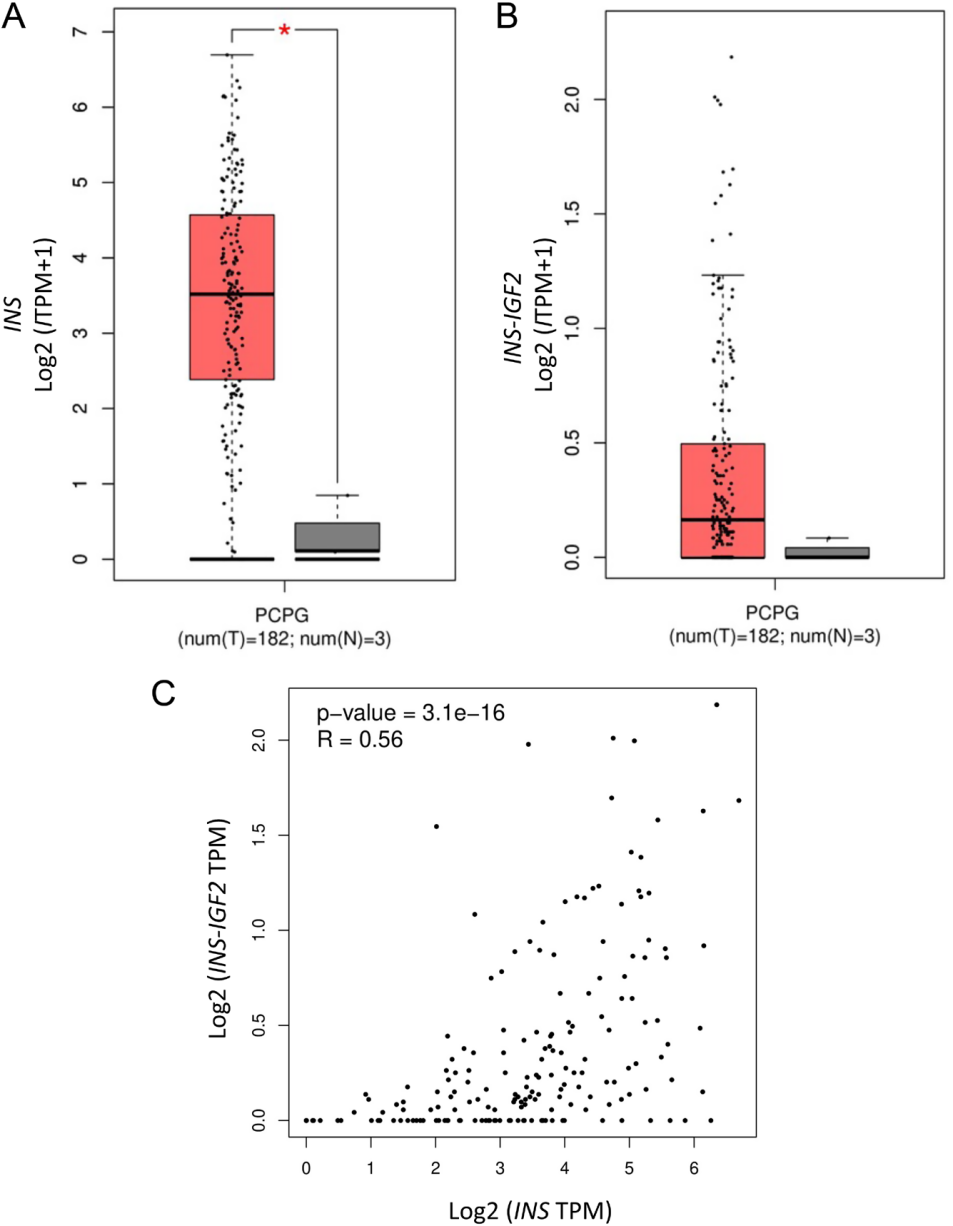
*INS* and *INS-IGF2* expression in phaeochromocytomas and paragangliomas (PCPG) (A). Expression of *INS* transcript is significantly elevated in PCPG (red) compared to normal tissue (grey) in the TCGA dataset after ANOVA analysis with a cut off value of 1.5 (*P* = 0.01). The expression data are first log_2_(TPM+1) transformed for differential analysis and the log_2_FC is defined as median (tumour) – median (normal). Genes with higher |log_2_FC| values and lower q values than pre-set thresholds are considered differentially expressed genes (B). Expression of *INS-IGF2* transcript analysed as in (A and C). Spearman correlation analysis of *INS* and *INS-IGF2* expression. The data corroborate our findings in the 20 phaeochromocytomas.

To validate the transcription results in a larger cohort, *INS and INS-IGF2* expressions were evaluated in TCGA cohort of phaeochromocytomas and paragangliomas. The analysis showed a significant difference (*P* = 0.01) in the expression of *INS* in the tumours compared to the normal adrenal gland (Fig. 5A). There was also an increase of *INS-IGF2* in tumours compared to normal tissue (Fig. 5B). Similar to our results, the *INS-IGF2* expression levels are lower than the *INS* expression levels. The degree of expression of the two transcripts correlates positively (Fig. 5C). Thus, the transcription data obtained from TCGA corroborate the results from our cohort.

In conclusion, activation of expression from the insulin locus is common in phaeochromocytomas. This opens the possibility that the production of insulin and the hybrid polypeptide translated from *INS-IGF2* transcript in the tumours may contribute both to autocrine tumour growth effects and the glucose imbalances seen in these patients.

## Discussion

We present evidence that most phaeochromocytomas contain cells that express *INS* transcript and *INS-IGF2* transcript 2. Staining with anti-insulin antibodies supports that the transcripts are translated to polypeptides. Our findings add to the previous knowledge about the biology of these tumours and their ectopic hormone production. This opens the possibilities that the overexpression may cause or contribute both to autocrine tumour growth effects and the two types of glucose imbalance seen in patients with a phaeochromocytoma. Our cohort of patients, recruited unselectively, constitutes a typical cohort, with two germline predisposing mutations. Other clinical features, presented in Supplementary Table 1, are also as expected in an unselected cohort.

In the fully developed body, only β-cells of the pancreas express insulin on both transcriptional and polypeptide levels. Our results of *INS* transcript levels close to the endogenous pancreatic levels, and the anti-insulin staining with an intracellular granular morphology (Fig. 4) similar to insulin-containing granule in *β*-cells in the pancreas, is, therefore, unexpected.

What could give phaeochromocytoma cells the ability to express insulin? Chromaffin cells and pancreatic *β*-cells are not closely related developmentally, as they come from neuronal (23) and epithelial (24) origin, respectively. However, data from studies of *β*-cell differentiation suggest that the developmental distance may be relatively short. First, the whole genomic area around the *INS* gene (from before the beginning of the *INS* gene and well into the *IGF2* gene) has open chromatin conformation (25), reflecting a permissive state as the first requirement for transcription. Secondly, during differentiation, *β*-cells acquire a methylation pattern more similar to neuronal cells than pancreatic acinar cells (24). This is interesting, as most phaeochromocytoma tumours show signs of tumour-specific changes in methylation status (26). Thirdly, demethylation of the insulin promotor, rendering it active, happens late in *β*-cell differentiation, and already before the activation of the promotor, low levels of *INS* transcripts can be detected (27). These three points support that low-level expression from the open chromatin in this region is possible without a fully active promotor.

Several lines of evidence further support the possibility of insulin expression in phaeochromocytomas. The *IGF2* gene is localised in a region that undergoes classical maternal–paternal imprinting. Overexpression of *IGF2* transcript is common in phaeochromocytomas, and mechanisms found so far are tumour- specific genomic changes in copy number, uni-parental disomy, and methylation (19). Amplifications resulting in several copies of the whole *INS*-to-*IGF2* locus would give more templates also for insulin transcription. The uni-parental disomy, where the mother’s imprinted/closed copy of *IGF2* has been exchanged to a second copy of the father’s actively transcribing copy, would also increase the possibility of transcription from the *INS-to-IGF2* locus. Lastly, as touched upon earlier, the methylation changes leading to active *IGF2* promotors may spill over to the closely located *INS* gene leading to its activation. Thus, there are several possible mechanisms by which genomic changes activating transcription from the *IGF2* gene also could increase the expression of the *INS* gene in phaeochromocytoma.

Expression of *INS-IGF2* is not common in normal adult tissue (28). In tumours, it has been found in non-small-cell lung cancer (29) and insulinomas (30). We find a high degree of correspondence between the expression of *INS* and *INS-IGF2* transcripts, which is expected as they are transcribed from the same promotor (28). The need for four more cycles to detect the *INS-IGF2* transcript suggests different expression levels, which is supported by the TCGA data showing about a 60-fold difference in expression (Fig. 5A and B).

Kamio *et al.* (3) already in 1991 found that five of seven phaeochromocytomas stained positively with anti-insulin antibodies. We now confirm their finding in a larger study, where 16 of 20 tumours stained positively for insulin. In addition, we show overexpression of the *INS* and *INS-IGF2* transcripts, and the 182 tumours in the TCGA cohort corroborate this finding, suggesting a common phenomenon.

The anti-insulin staining strongly suggests that insulin is produced by these tumours.

In addition, the *INS-IGF2* transcript 2 encodes a 200 amino acid protein starting with the preproinsulin signal peptide, the insulin B chain, and the first eight amino acids in the proinsulin C-peptide (28). If the B chain of *INS-IGF2* polypeptide retains the same helical configuration as in insulin, it may contain epitopes present on insulin and thereby binding the same antibody. Supporting evidence comes from the finding that anti-insulin autoantibodies from patients with diabetes type 1 recognise epitopes on the B-chain part of the peptide translated from the *INS-IGF2* transcript 2 (31). Thus, both insulin and the hybrid polypeptide may stain with the anti-insulin antibody.

The expression analyses and the immunostaining show that the expression levels vary substantially between the phaeochromocytomas. The clonal expansion in one sample and the clusters of positive cells in some others indicate a potential for further clonal expansion revealing variations also within tumours. Such tumour heterogeneity is well known in both cancers and benign tumours, including phaeochromocytomas (32). Another example is aldosterone-producing tumours from the adrenal cortex (33) showing clonal distributions very similar to what we find in tumours from the medulla. Therefore, the few negative tumours in our cohort may express *INS* and *INS-IGF2* transcripts and corresponding polypeptides in other areas than we examined.

We found no correlation between the degree of immunostaining and the expression of the transcripts. Several mechanisms could explain this lack of correlation. Differences in synthesis and modulation of a protein’s half-life are possible mechanisms (34). For example, it has been reported that *INS* transcript 1 gives more efficient translation into protein than transcripts 2–4 (35). Based on the granules we see in the insulin staining, another possible way to change the relationship between transcripts and polypeptides is the secretion kinetics of the final polypeptide product. Rapid secretion would lead to small amounts present in the cell whereas cells without secretion would retain products inside the cells and, therefore, stain more strongly.

The activation of a wider region around the insulin gene may have possible autocrine stimulatory growth effects in phaeochromocytomas. They overexpress IGF-2 and its receptor (20) and the IGF-1 receptor (36), and the overexpression is most pronounced in malignant tumours (37). Our finding of overexpression of insulin may also induce autocrine growth stimulation. This is, however, less likely, because highly supraphysiological insulin concentrations are required to induce growth (38).

Phaeochromocytomas induce two types of glucose imbalances, hyperglycaemia and hypoglycaemic attacks, both occurring on top of the typical phaeochromocytoma symptoms and signs. Hyperglycaemia is common and is probably caused by two effects of catecholamines, namely increased insulin resistance and inhibition of insulin secretion from beta cells (14). Hypoglycaemic attacks are rare, only a few cases have been reported (15, 16, 17, 18) and the mechanism is not known. It has been postulated (16) that catecholamine induced *β*-adrenoceptor- mediated release of insulin from pancreatic *β*-cells may override the prevailing *α*-adrenoceptor mediated inhibition of release and the insulin resistance seen in most phaeochromocytoma patients (14). However, no evidence for this overriding mechanism is presented, and it seems unlikely that in just a few rare patients the overwhelming hyperglycaemic catecholamine effects should be switched to the opposite. Empty glucose stores in the liver have also been suggested to contribute (16). However, other phaeochromocytoma patients experience recurrent postprandial reactive hypoglycaemic attacks without any malnutrition (17, 18).

It is of considerable interest that our findings suggest new contributing mechanisms for both types of glucose imbalances. Obviously, hypersecretion of insulin could cause hypoglycaemic attacks. However, only very few patients suffer from hypoglycaemia and most of our phaeochromocytomas stain positively. Therefore, only tumours with heavy staining and clonal expansion of such cells may contain sufficient amounts of insulin. In addition, to cause hypoglycaemia, the normal control that stops insulin secretion from *β*-cells when blood glucose is low must be defective. Generally, tumours with ectopic hormone production often lack a normal regulatory mechanism of hormone secretion (39). This is also shown for insulin-producing tumours causing hypoglycaemia (10, 35). Therefore, if a phaeochromocytoma produces much insulin, from the whole tumour or a local clone, and the normal regulatory brake on secretion of insulin to the blood is defect, the tumour could cause hypoglycaemia. Normal β-cells constitute only about 2% of pancreatic cells (40, 41) so a phaeochromocytoma of around 5 cm would contain a sufficient number of insulin-producing cells.

The few cases that reported reactive hypoglycaemia in phaeochromocytoma suggest that these tumours may have a trigger for insulin secretion. In the pancreas, one of many triggers for insulin secretion is the stimulation of glucagon-like peptide 1 receptors (GLP1R). GLP1R expression/protein has been found on phaeochromocytoma cells (42) and tumours (43), but whether they function normally or if they are expressed on cells positive for anti-insulin staining is not known.

Our findings may also suggest a contributing mechanism for the more common hyperglycaemia seen in phaeochromocytoma patients. The *INS-IGF2* polypeptide, if secreted, may block insulin receptors, causing insulin resistance with hyperglycaemia. Normally, insulin stimulates its receptor by binding to two different sites on the receptor. The major site binds the insulin B-chain (44). The hybrid contains the B-chain. If this B-chain part retains the same helical configuration as in insulin, the hybrid could bind to only one of the two sites, and, therefore, block the receptor.

In sum, our data suggest possible mechanisms that may contribute to dysregulated glucose metabolism in phaeochromocytoma that can be addressed in future studies. First, it should be demonstrated whether the anti-insulin staining granule structures we see are fully functional with the secretion of the polypeptide products or whether secretion requires additional triggers. One interesting question that remains to be explored is to define the mechanism(s) explaining the few cases of reactive hypoglycaemia reported in the literature given that insulin is commonly expressed in tumours, but hypoglycaemia cases are rare. As alluded to above, it remains to be defined whether the hybrid polypeptide binds to the antibodies and may compete with insulin for insulin-receptor binding and thereby contribute to hyperglycaemia. Detailed biochemical studies of receptor-binding kinetics may elucidate this point. The clonal expansions imply that heterogeneity within a tumour must be considered. Finally, but perhaps most importantly, future studies must be performed in order to correlate our findings with clinical and genetic aspects on a larger cohort of phaeochromocytoma patients.

## Conclusion

Most phaeochromocytomas contain cells that overexpress *INS* and *INS-IGF2* transcripts. Most tumours also display heterogeneous expression of polypeptides immunoreactive to monoclonal anti-insulin antibodies. Clinically this may relate to both hyperglycaemia and hypoglycaemic attacks seen in patients with phaeochromocytoma as well as autocrine tumour growth.

## Supplementary Material

Supplementary Table 1. Patient characteristics

## Declaration of interest

The authors declare that there is no conflict of interest that could be perceived as prejudicing the impartiality of the research reported.

## Funding

The study was supported by South-Eastern Regional Infrastructure for Clinical and Translational Research (SERIT) grant number 276984.

## References

[1] Dimitriadis GK Angelousi A Weickert MO Randeva HS Kaltsas G Grossman A. Paraneoplastic endocrine syndromes. Endocrine-Related Cancer 2017 24 R173–R190. (10.1530/ERC-17-0036) 28341725

[2] Ilias I Torpy DJ Pacak K Mullen N Wesley RA Nieman LK. Cushing’s syndrome due to ectopic corticotropin secretion: twenty years’ experience at the National Institutes of Health. Journal of Clinical Endocrinology and Metabolism 2005 90 4955–4962. (10.1210/jc.2004-2527) 15914534

[3] Kamio T Shigematsu K Kawai K Tsuchiyama H. Immunoreactivity and receptor expression of insulinlike growth factor I and insulin in human adrenal tumors. An immunohistochemical study of 94 cases. American Journal of Pathology 1991 138 83–91. PMC1886050 1702931

[4] Stagno PA Petras RE Hart WR. Strumal carcinoids of the ovary. An immunohistologic and ultrastructural study. Archives of Pathology and Laboratory Medicine 1987 111 440–446. 3551874

[5] Leach SD LaMorte AI True LD Flynn SD Schwartz PE Cahow CE Kinder BK. Aberrant hormone production from ovarian neoplasms: strategies for diagnosis and therapy. World Journal of Surgery 1990 14 335–341. (10.1007/BF01658520) 2164282

[6] Ashton MA Strumal carcinoid of the ovary associated with hyperinsulinaemic hypoglycaemia and cutaneous melanosis. Histopathology 1995 27 463–467. (10.1111/j.1365-2559.1995.tb00311.x) 8575738

[7] Morken NH Majak B Kahn JA. Insulin producing primary ovarian carcinoid tumor. Acta Obstetricia et Gynecologica Scandinavica 2007 86 500–501. (10.1080/00016340600613477) 17486477

[8] Seckl MJ Mulholland PJ Bishop AE Teale JD Hales CN Glaser M Watkins S Seckl JR. Hypoglycemia due to an insulin-secreting small-cell carcinoma of the cervix. New England Journal of Medicine 1999 341 733–736. (10.1056/NEJM199909023411004) 10471459

[9] Shames JM Dhurandhar NR Blackard WG. Insulin-secreting bronchial carcinoid tumor with widespread metastases. American Journal of Medicine 1968 44 632–637. (10.1016/0002-9343(6890065-x) 4296076

[10] Battocchio M Zatelli MC Chiarelli S Trento M Ambrosio MR Pasquali C De Carlo E Dassie F Mioni R Rebellato A Ovarian tumors secreting insulin. Endocrine 2015 49 611–619. (10.1007/s12020-015-0605-y) 25896552

[11] Morgello S Schwartz E Horwith M King ME Gorden P Alonso DR. Ectopic insulin production by a primary ovarian carcinoid. Cancer 1988 61 800–805. (https://doi.org/10.1002/1097-0142(19880215)61:4<800::aid-cncr2820610426>3.0.co;2-3) 3276388 10.1002/1097-0142(19880215)61:4<800::aid-cncr2820610426>3.0.co;2-3

[12] Ramkumar S Dhingra A Jyotsna V Ganie MA Das CJ Seth A Sharma MC Bal CS. Ectopic insulin secreting neuroendocrine tumor of kidney with recurrent hypoglycemia: a diagnostic dilemma. BMC Endocrine Disorders 2014 14 36. (10.1186/1472-6823-14-36) PMC4046058 24741994

[13] Uysal M Temiz S Gul N Yarman S Tanakol R Kapran Y. Hypoglycemia due to ectopic release of insulin from a paraganglioma. Hormone Research 2007 67 292–295. (10.1159/000099291) 17284922

[14] Erlic Z Beuschlein F. Metabolic alterations in patients with pheochromocytoma. Experimental and Clinical Endocrinology and Diabetes 2019 127 129–136. (10.1055/a-0649-0960) 30011405

[15] Abdulhadi B Anastasopoulou C Lekprasert P. Tumor-induced hypoglycemia: an unusual case report and review of literature. AACE Clinical Case Reports 2021 7 80–83. (10.1016/j.aace.2020.11.002) 33851027 PMC7924146

[16] Frankton S Baithun S Husain E Davis K Grossman AB. Phaeochromocytoma crisis presenting with profound hypoglycaemia and subsequent hypertension. Hormones 2009 8 65–70. (10.14310/horm.2002.1224) 19269923

[17] Følling I Olsen AL Nermoen I Thorsby PM. Phaeochromocytoma and hypoglycaemic fits: a case report. Endocrine Abstracts 2015 37 EP1156. (10.1530/endoabs.37.EP1156)

[18] Thonangi RP Bhardwaj M Kulshreshtha B. A case report of reactive hypoglycemia in a patient with pheochromocytoma and it’s review of literature. Indian Journal of Endocrinology and Metabolism 2014 18 234–237. (10.4103/2230-8210.129120) 24741525 PMC3987279

[19] Nielsen HM How-Kit A Guerin C Castinetti F Vollan HK De Micco C Daunay A Taieb D Van Loo P Besse C Copy number variations alter methylation and parallel IGF2 overexpression in adrenal tumors. Endocrine-Related Cancer 2015 22 953–967. (10.1530/ERC-15-0086) 26400872 PMC4621769

[20] Gelato MC Vassalotti J. Insulin-like growth factor-II: possible local growth factor in pheochromocytoma. Journal of Clinical Endocrinology and Metabolism 1990 71 1168–1174. (10.1210/jcem-71-5-1168) 2172273

[21] Tang Z Li C Kang B Gao G Li C Zhang Z. GEPIA: a web server for cancer and normal gene expression profiling and interactive analyses. Nucleic Acids Research 2017 45 W98–W102. (10.1093/nar/gkx247) PMC5570223 28407145

[22] Gill AJ Succinate dehydrogenase (SDH)-deficient neoplasia. Histopathology 2018 72 106–116. (10.1111/his.13277) 29239034

[23] Aunis D Exocytosis in chromaffin cells of the adrenal medulla. International Review of Cytology 1998 181 213–320. (10.1016/s0074-7696(0860419-2) 9522458

[24] Bramswig NC Kaestner KH. Organogenesis and functional genomics of the endocrine pancreas. Cellular and Molecular Life Sciences 2012 69 2109–2123. (10.1007/s00018-011-0915-z) 22241333 PMC3340510

[25] Kuroda A Rauch TA Todorov I Ku HT Al-Abdullah IH Kandeel F Mullen Y Pfeifer GP Ferreri K. Insulin gene expression is regulated by DNA methylation. PLoS ONE 2009 4 e6953. (10.1371/journal.pone.0006953) 19742322 PMC2735004

[26] Castro-Vega LJ Lepoutre-Lussey C Gimenez-Roqueplo AP Favier J. Rethinking pheochromocytomas and paragangliomas from a genomic perspective. Oncogene 2016 35 1080–1089. (10.1038/onc.2015.172) 26028031

[27] Ku HT Chai J Kim YJ White P Purohit-Ghelani S Kaestner KH Bromberg JS. Insulin-expressing colonies developed from murine embryonic stem cell-derived progenitors. Diabetes 2007 56 921–929. (10.2337/db06-0468) 17395739

[28] Monk D Sanches R Arnaud P Apostolidou S Hills FA Abu-Amero S Murrell A Friess H Reik W Stanier P Imprinting of IGF2 P0 transcript and novel alternatively spliced INS-IGF2 isoforms show differences between mouse and human. Human Molecular Genetics 2006 15 1259–1269. (10.1093/hmg/ddl041) 16531418

[29] Gao S Lin Z Li C Wang Y Yang L Zou B Chen J Li J Feng D Song Z lncINS-IGF2 promotes cell proliferation and migration by promoting G1/s transition in lung cancer. Technology in Cancer Research and Treatment 2019 18 1533033818823029. (10.1177/1533033818823029) 30803359 PMC6374000

[30] Johannessen LE Panagopoulos I Haugvik SP Gladhaug IP Heim S Micci F. Upregulation of INS-IGF2 read-through expression and identification of a novel INS-IGF2 splice variant in insulinomas. Oncology Reports 2016 36 2653–2662. (10.3892/or.2016.5132) 27667266

[31] Kanatsuna N Taneera J Vaziri-Sani F Wierup N Larsson HE Delli A Skärstrand H Balhuizen A Bennet H Steiner DF Autoimmunity against INS-IGF2 protein expressed in human pancreatic islets. Journal of Biological Chemistry 2013 288 29013–29023. (10.1074/jbc.M113.478222) PMC3789998 23935095

[32] Crona J Backman S Maharjan R Mayrhofer M Stalberg P Isaksson A Hellman P Bjorklund P. Spatiotemporal heterogeneity characterizes the genetic landscape of pheochromocytoma and defines early events in tumorigenesis. Clinical Cancer Research 2015 21 4451–4460. (10.1158/1078-0432.CCR-14-2854) 25991818

[33] Dekkers T ter Meer M Lenders JW Hermus AR Schultze Kool L Langenhuijsen JF Nishimoto K Ogishima T Mukai K Azizan EA Adrenal nodularity and somatic mutations in primary aldosteronism: one node is the culprit? Journal of Clinical Endocrinology and Metabolism 2014 99 E1341–E1351. (10.1210/jc.2013-4255) 24758183

[34] Liu Y Beyer A Aebersold R. On the dependency of cellular protein levels on mRNA abundance. Cell 2016 165 535–550. (10.1016/j.cell.2016.03.014) 27104977

[35] Minn AH Kayton M Lorang D Hoffmann SC Harlan DM Libutti SK Shalev A. Insulinomas and expression of an insulin splice variant. Lancet 2004 363 363–367. (10.1016/S0140-6736(0415438-X) 15070567

[36] Fottner C Minnemann T Kalmbach S Weber MM. Overexpression of the insulin-like growth factor I receptor in human pheochromocytomas. Journal of Molecular Endocrinology 2006 36 279–287. (10.1677/jme.1.01975) 16595699

[37] Fernandez MC Martin A Venara M Calcagno Mde L Sanso G Quintana S Chemes HE Barontini M Pennisi PA. Overexpression of the insulin-like growth factor 1 receptor (IGF-1R) is associated with malignancy in familial pheochromocytomas and paragangliomas. Clinical Endocrinology 2013 79 623–630.(10.1111/cen.12205) 23506534

[38] Bedinger DH Adams SH. Metabolic, anabolic, and mitogenic insulin responses: a tissue-specific perspective for insulin receptor activators. Molecular and Cellular Endocrinology 2015 415 143–156. (10.1016/j.mce.2015.08.013) 26277398

[39] Kaltsas G Androulakis II de Herder WW Grossman AB. Paraneoplastic syndromes secondary to neuroendocrine tumours. Endocrine-Related Cancer 2010 17 R173–R193. (10.1677/ERC-10-0024) 20530594

[40] Robertson FO A quantitative estimation of the pancreatic islet tissue. QJM 1937 6 287–300.(10.1093/oxfordjournals.qjmed.a068286)

[41] Saisho Y Butler AE Manesso E Elashoff D Rizza RA Butler PC. β-Cell mass and turnover in humans: effects of obesity and aging. Diabetes Care 2013 36 111–117. (10.2337/dc12-0421) 22875233 PMC3526241

[42] Saber-Ayad M Zaher D Manzoor S Omar H. PO-453 effect of GLP-1 on proliferation and migration in pheochromocytoma and colorectal cancer cells. ESMO Open 2018 3 A199–A200. (10.1136/esmoopen-2018-EACR25.474)

[43] Körner M Stöckli M Waser B Reubi JC. GLP-1 receptor expression in human tumors and human normal tissues: potential for in vivo targeting. Journal of Nuclear Medicine 2007 48 736–743. (10.2967/jnumed.106.038679) 17475961

[44] Thorsøe KS Schlein M Steensgaard DB Brandt J Schluckebier G Naver H. Kinetic evidence for the sequential association of insulin binding sites 1 and 2 to the insulin receptor and the influence of receptor isoform. Biochemistry 2010 49 6234–6246. (10.1021/bi1000118) 20568733

