# Supplementary material for: Phaeochromocytomas overexpress insulin transcript and produce insulin
Source: Endocr Connect. 2021 Jun 25;10(8):815–24. doi: 10.1530/EC-21-0269 (PMC8346199; doi:10.1530/EC-21-0269)
Supplement: Supplementary Table 1. Patient characteristics [file supplementary_table_1.pdf]

Supplementary Table 1. Patient characteristics

Patient	Gender/age	Caucasian	Hyperglycaemia*	Tumour size (cm)	SDHB staining** Tumour	Normal
1	F/64	+	+	5.2	+	
2	F/32	+	-	7.5	+	
3	M/58	+	+	9.5	+	
4	F/50	+	+	6.0	+	
5	M/56	-	-	3.5	+	
6	F/67	+	-	4.3	+	
7	F/42	-	+	2.5	+	
8	M/69	+	-	5.5	+	
9	M/45	+	-	5.0	+	
10	F/55	-	-	3.0	+	
11	F/70	+	+	2.8	+	
12	F/50	+	-	5.0	+	
13	F/53	+	+	4.0	+	
14	M/59	+	-	5.0	+	
15	F/51	+	-	1.9	(+)	+
16	M/43	+	-	2.3	(+)	+
17	M/64	+	-	2.0	+	
18	M/41	+	-	9.0	(+)	+
19	M/36	+	+	9.5	+	
20	M/30	+	-	6.8	+	

* Hyperglycaemia + defined as known diabetes 2 or HbA1c ≥ 48 mmol/mol or fasting blood glucose ≥ 7.0 mmol/l or nonfasting value ≥ 11.1.

** SDHB stain (+) means somewhat weak staining, with staining of the normal adrenal tissue shown, indicating the presence of succinate dehydrogenase B.
